# Faculty of Radiation Oncology 2022 Workforce Census

**DOI:** 10.1111/1754-9485.13883

**Published:** 2025-08-07

**Authors:** Hon Trinh, Nathan Stevens, Gerard Adams, Raphael Chee, Tuan Ha, Marcel Knesl, Jack Mitchell, Sakshi Nagpal, Edward Sia, Daniel Xing, the RANZCR Economics and Workforce Committee

**Affiliations:** ^1^ NSW Health; Illawarra Shoalhaven Cancer and Haematology Network Nowra Australia; ^2^ University of Wollongong Wollongong Australia; ^3^ Royal Australian and New Zealand College of Radiologists Wellington New Zealand; ^4^ Genesis Care Sydney Australia; ^5^ Rural Clinical School University of Queensland Bundaberg Australia; ^6^ School of Medical & Health Sciences Edith Cowan University Perth Australia; ^7^ Queensland Health; Sunshine Coast University Hospital Birtinya Queensland Australia; ^8^ Icon Group Brisbane Australia; ^9^ Griffith University School of Medicine Queensland Australia; ^10^ Queensland Health; Royal Brisbane and Women's Hospital Herston Queensland Australia; ^11^ Queensland Health; Townsville University Hospital Douglas Queensland Australia

**Keywords:** Australia, census, census data, New Zealand, radiation oncology, workforce

## Abstract

**Introduction:**

This paper reports the key findings of the Faculty of Radiation Oncology 2022 workforce census. This is the first census since the COVID‐19 pandemic and questions have been updated to assess the impact on RANZCR trainees and fellows. This report focuses on the analysis of respondents from Australia, New Zealand and overseas members, with a separate paper to follow focusing exclusively on New Zealand respondents.

**Method:**

The census was conducted in mid‐late 2022 with many questions repeated from previous censuses. New questions were asked about theranostics, working remotely, hypofractionation and the impact of COVID‐19 on work practices.

**Results:**

The census was sent to 591 radiation oncologists with an overall response rate of 52%. Almost half of respondents (*n* = 94/210; 45%) indicated that COVID‐19 had no impact on the uptake of hypofractionation. Hypofractionation was most used by respondents in breast and prostate treatment (*n* = 134/200; 67% and *n* = 112/194; 58% respectively). Five respondents (*n* = 5/270; 2%) currently practise in theranostics, with the majority treating thyroid cancers within the public sector.

Just under half (*n* = 81/167; 49%) of invited trainees responded. The majority felt that COVID‐19 had a negative impact on their training. There has been a decrease in the number of new fellows seeking to complete further fellowships. Employment remains at very high levels for new fellows ( > 98%).

**Conclusion:**

The impact of COVID‐19 on local practices and workloads was not as significant as seen overseas. There continues to be an increasing trend of radiation oncologists working in the private sector. The lack of indigenous representation within our profession continues to be an area that needs further attention.

## 1 Introduction

This is the seventh Royal Australian and New Zealand College of Radiologists (RANZCR) workforce census of radiation oncologists and trainees in Australia, New Zealand and Singapore, with previous studies undertaken in 1996, 2000, 2006, 2010, 2014 and 2018 [1, 2, 3, 4, 5, 6]. The census is conducted every 4 years, with this being the first census since the COVID‐19 pandemic, which triggered unprecedented changes to health services globally. Several reviews and meta‐analyses have shown that a substantial proportion of healthcare workers experienced clinically significant levels of psychological distress during the pandemic [7].

The information obtained in this census will be used to analyse workforce trends and predict future staffing and training requirements. The actual census questions are included in Appendix I.

## 2 Methods

The 2022 workforce census was conducted from June to October 2022. All active radiation oncologists and trainees listed on the FRANZCR database were invited to participate. Questions were designed and tested by members of the Economics and Workforce Committee (EWC), which comprises 12 members, as well as 3 college secretariat staff. A key purpose of the census was to analyse workforce trends, and many of the questions were repeated from the 2014 and 2018 census. It was also considered relevant to question the membership about new trends and issues that are topical. For radiation oncologists, new questions were asked about theranostics, working remotely, the use of hypofractionation for different tumour types, the impact of COVID‐19 on work practices and the practice of brachytherapy outside of gynae‐oncology. There was a separate section for questions related to the impact of COVID‐19 on trainees.

The census was distributed via Survey Monkey with weekly email reminders. It was closed at the end of October 2022. Some respondents did not answer every question, and there were also certain logical rules that resulted in a different number of respondents between questions. For example, for radiation oncologists who identified themselves as retired, questions regarding work hours or workplace were omitted. The responses were analysed to highlight workforce trends. The analysis was done with Microsoft Excel.

## 3 Results

### 3.1 Radiation Oncologists

#### 3.1.1 Eligible Study Sample

All radiation oncologists and trainees registered on the RANZCR database as of June 2020 were eligible. This included those retired, working overseas and educational affiliates.

The census was sent to 591 radiation oncologists and trainees with responses from 308 (52%). 18 radiation oncologists identified themselves as retired and were not asked questions relevant to practising radiation oncologists.

An analysis of non‐respondents revealed similar demographics to respondents, and thus, the results were not influenced by non‐responder bias.

#### 3.1.2 Personal and Demographic Data

The RANZCR database identified *n* = 346/593; 58% of radiation oncologists as male and *n* = 245/593; 41% as female. The average age of consultant radiation oncologists was 51.0 years, with a median of 49 years (range 32–91). Most radiation oncologist respondents were from New South Wales (*n* = 80/307; 26%) followed by Queensland (*n* = 60/307; 19%) and then Victoria (*n* = 57/307; 19%). Comparatively, the RANZCR membership database shows that in 2022, *n* = 169/593; 28% of radiation oncologists are from New South Wales, *n* = 101/593; 17% from Queensland, and *n* = 123/593; 21% from Victoria.

Respondents were asked to indicate the ethnicity to which they most identified. The majority of respondents identified as either Australian (*n* = 138/296; 47%) or New Zealanders (*n* = 30/296; 11%). A substantial percentage identified as Asian (Chinese, Southeast Asian or other Asian) (*n* = 63/296; 21%) and Indian (*n* = 23/296; 8%). There were no respondents who identified as Aboriginal or Torres Strait Islander in this census.

On average, responding radiation oncologists graduated from medical school 25 years ago. Of the 273 respondents, well over half (*n* = 176/273; 65%) graduated between 1990 and 2009.20 per cent (*n* = 62/307) of respondents held another qualification besides their primary medical degree and fellowship with *n* = 20/62; 32%, holding a Doctor of Medicine (M.D.), *n* = 22/62; 35%, a Doctor of Philosophy (Ph.D.), *n* = 13/62; 21% a Master of Philosophy (M.Phil.) and *n* = 4/62; 6% a Doctor of Medical Science (D. Med. Sc). The remaining 5% was not specified by respondents.

#### 3.1.3 Special Interest Areas and Generalist vs. Subspecialist

**FIGURE 1:**
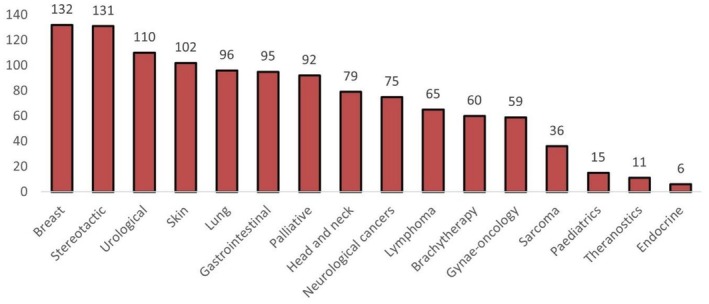
Reported special interest areas.

Respondents were asked to identify subspecialty interests in their practice (Figure 1). There were 16 in the ‘other’ category including benign disease, MR Linac, artificial intelligence, global oncology, neurological diseases and medical information. Respondents were asked to identify how much time was spent on brachytherapy; 49/307; 16% of the workforce spent at least some time on brachytherapy. Of these, 32/207; 10% practised in gynae‐oncology, 10/307; 3%, and 15/307; 5% performed LDR and HDR brachytherapy respectively.

Most respondents indicated they were subspecialists (*n* = 133/210, 63%) compared to generalists (*n* = 77/210, 37%).

#### 3.1.4 Leadership Positions

Most respondents (*n* = 165/286; 62%) held at least one leadership position. The number of leadership positions held per respondent ranged from one to nine (Table 1).

**TABLE 1:** Leadership positions held.

Number of leadership positions	Male (n)	Female (n)	Total positions held (n)
0	58	45	103
1	42	38	80
2	26	21	47
3	13	10	23
4	3	3	6
5	2	2	4
6	2	0	2
7	1	0	1
8	0	1	1
9	0	1	1
Total	147	121	268

#### 3.1.5 Practice Location and Work Hours

Respondents worked at an average of 2.1 sites, with a median of 2.0 (range 1–7). Most (*n* = 114/243; 47%) worked exclusively in the public sector. Over one‐third (*n* = 97/243; 40%) worked across public and private sectors, and a minority (*n* = 32/243; 13%) worked exclusively in private.

#### 3.1.6 Work Hours

Total number of hours worked (onsite and offsite) and the clinical hours spent each week is reported in Table 2. Mean FTE status was 0.96 (median 1.0; range 0.2–2.0) with males averaging 1.0 FTE and females 0.9 FTE. Males reported longer actual and clinical hours per week compared to females, averaging 45.0 h vs. 42.9 h (median 43 h vs. 42 h) and 37.4 h vs. 32.2 h (median 37 h vs. 32 h) respectively.

**TABLE 2:** Reported total and actual clinical hours per week by location.

	Actual Hours (onsite + offsite)		Clinical Hours	
	Mean	Range	Mean	Range
ACT	43.4	32–65	37.0	29–46
NSW	40.8	5–68	31.1	4–96
QLD	42.0	12–70	36.3	10–85
SA	39.8	22–62	30.3	20–39
TAS	49.0	43–55	46.0	37–55
VIC	43.2	18–80	33.4	4–78
WA	52.5	30–71	45.3	32–57
NZ	50.4	25–74	37.9	8–69
Overseas	46.5	25–64	38.5	23–53
Total	44.1	5–80	35	4–96

Respondents working exclusively in the private sector averaged 40.8 h, those exclusively working in the public sector averaged 43.0 h and those working in public/private practice averaged 46.4 h per week.

Western Australians worked the longest actual hours per week (52.5 h), followed by respondents from New Zealand (50.4 h) whilst Tasmanians averaged the longest clinical hours at 46.0 h per week. However, most states shared a close range in actual and clinical hours. This may reflect the number of radiation oncologists in each state per 100,000 population (WA 1.0 RO per 100,000 population; NZ 1.2 per 100,000, NSW 1.9 per 100,000 and Tasmania 1.6 per 100,000 according to RANZCR membership data compared against ABS population statistics from 2022).

#### 3.1.7 New Cases, Follow‐Ups, Treatment Reviews and Planning Hours

Radiation oncologists spent an average of 6.4 h (median: 6 h) per week on new cases, 8.8 h (median: 8 h) on follow‐ups, and 3.8 h (median: 3 h) on treatment reviews.

Those in the private sector spent more hours on treatment reviews with 5.0 h vs. 4.0 h for those in public/private and 3.2 h for those in the public sector.

**FIGURE 2:**
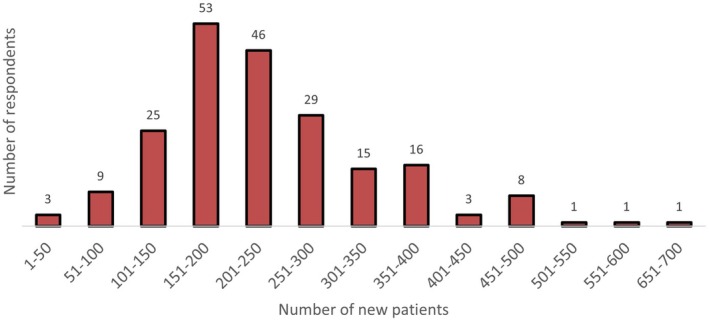
New patients consulted in a year.

Figure 2 shows the number of new consultations per radiation oncologist per year. Respondents averaged 49.7 min (m) (median = 50 m) for each new case, 17.9 m (median: 20 m) per follow‐up case and 10.5 m (median: 10 m) per treatment review.

For follow‐ups, those in the public sector spend the most time with 18.9 m vs. 15.6 m for those in the private sector and 17.5 m for those in public/private.

Women averaged 10.9 m per treatment review compared with 10.2 m for men. A breakdown for all radiation oncologists can be seen in Table 3.

**TABLE 3:** Hours spent per week on activities.

	Mean (hours)	Median (hours)	Range (hours)
New cases	6.4	6.0	2–20
Follow up cases	8.8	8.0	1–25
On treatment’ reviews	3.8	3.0	1–12
Simulation (hours per week)	1.8	1.0	0–6
Dosimetry (hours per week)	2.4	2.0	0–10
Contouring (hours per week)	6.5	6.0	1–25
Supervision of registrars (hours per week)	4.6	3.0	0–40
Research and trials (hours per week)	2.3	1.0	0–16
Practice management (hours per week)	2.8	2.0	0–30
Academic teaching (hours per week)	1.2	1.0	0–20
Jurisdiction/quality committees (hours per week)	1.3	1.0	0–10
Other (hours per week)	4.3	1.0	0–40

#### 3.1.8 Supervision, Teaching, Research and Other Hours

Radiation oncologists averaged 4.6 h (median: 3 h) on supervision, 2.3 h (median: 1 h) on research, 2.8 h (median: 2 h) on management, 1.2 h (median: 1 h) on teaching, 1.3 h (median: 1 h) on jurisdiction/quality committee and 4.3 h (median: 1 h) on ‘other’ activities per week. The ‘other’ category included college activities, professional education delivery, community public speaking, journal reading, administrative work, and answering emails.

#### 3.1.9 Hypofractionation and SBRT/SRS/SRT

SBRT was used by most respondents (*n* = 156/210; 74%) with the most common sites being lung and bone. Brain SRS/SRT was used by just under half of respondents (*n* = 101/210; 48%).

Respondents reported their uptake of hypofractionation across various procedures using a Likert scale (Always, most times, half the time, sometimes, no and N/A) (Table 4).

**TABLE 4:** Uptake of hypofractionation by procedure type.

Procedure	Always	Most times	Half the time	Sometimes	No	N/A	Total responses (n)
Breast (15/16 fractions)	53	62	5	14	1	66	200
Breast (5 fractions)	7	12	8	66	36	69	198
GBM (elderly 15 fractions)	25	45	9	18	3	100	200
Preoperative rectum (5 fractions)	2	12	13	72	7	91	197
Prostate (20 fractions) – excluding ultra hypofractionation (e.g., 7 or less)	33	50	16	13	2	80	194
Single fraction for bone metastases – excluding SBRT	27	69	45	54	6	6	207
Curative intent lung cancer (excluding SBRT) (e.g., 20 fraction schedule)	8	19	11	53	13	96	200

#### 3.1.10 Remuneration

Table 5 shows remuneration types across a range of demographics. When compared by sector type, there is a notable preference for incentive‐based income in the private sector (*n* = 21/29; 72%). This is also seen with a preference for a mixed income in respondents working across Public/Private (*n* = 61/86; 71% for mixed income). In the public sector, *n* = 78/95; 82% are on a fixed income.

**TABLE 5:** Remuneration type by sector and gender.

	Total Cohort		Female		Male	
Income Type	*n*	%	*n*	%	*n*	%
Fixed	100	48%	54	56%	46	40%
Incentive Based	14	7%	4	4%	10	9%
Mixed	96	46%	38	40%	58	51%
Total	210	100%	96	100%	114	100%

	Public		Public/Private		Private	
	*n*	%	*n*	%	*n*	%
Fixed	78	82%	17	20%	5	17%
Mixed	14	15%	61	71%	3	10%
Incentive	3	3%	8	9%	21	72%
Total	95	100%	86	100%	29	100%

#### 3.1.11 Employment and Future Practice

Of employed respondents (*n* = 147/210, 70%) indicated no intention to change their work hours over the next 3 years. A small number of respondents (3/279; 1%) reported being unemployed at the time of the census. One out of three intended to return to work within a year.

46% (*n* = 96/210) of the respondents had no intention to retire in the foreseeable future, but a small minority (*n* = 25/210, 12%) intended to retire within 5 years. Over a quarter of respondents (*n* = 55/210; 26%) intended to decrease hours, with 4% (*n* = 8/210) intending to increase hours.

19 retired members retired between the ages of 52 and 91.

#### 3.1.12 Early Career

There was a period of unemployment for *n* = 4/307 (1%) respondents after admission to fellowship. Most respondents became a consultant (*n* = 119/307, 39%) or fellow (*n* = 110, 39.7%) or engaged in locum work (*n* = 44, 16%). The remaining *n* = 30/307 (30%) did not give a response.

#### 3.1.13 Impact of COVID‐19

Respondents commented on the impact of COVID‐19 on their working habits, with 32% (*n* = 99/307) suggesting that their personal work patterns remained the same and 30% (*n* = 91/307) spending more time working from home but managing the same workload. Nearly one fifth (*n* = 59/307; 19%) increased workload due to unavailable colleagues.

Just over half of respondents (*n* = 108/210; 51%) agreed that COVID‐19 drove their adoption of hypofractionation ‘a little’. In a follow up question, most respondents (*n* = 112/210; 53%) acknowledged that they will continue to increase hypofractionation regardless of COVID‐19.

43% (*n* = 94/218) noted an increase in workload due to COVID‐19. The mean increase was 4.4%, ranging from −40% to +50%. Most respondents indicated waiting times have remained the same (*n* = 169/233; 73%). Other changes due to COVID‐19 include an increase in virtual multidisciplinary team meetings, telehealth/work from home, and a reduction in patient contact.

### 3.2 Trainees

#### 3.2.1 Eligible Study Sample and Demographics

167 trainees were identified in the RANZCR membership database with an even distribution of gender (men: *n* = 86, 51.5%; women: *n* = 81, 48.5%). There were *n* = 81/167 (48%) trainee respondents to the census; 48% (*n* = 39/81) were female.

Mean age of trainees was 34 years (median: 34, range: 26–52). The geographical origin reflected that of the invited radiation oncologists, with most trainees from New South Wales (*n* = 28/81; 35%), followed by New Zealand (*n* = 24/81; 30%), Queensland (*n* = 12/81; 15%) and Victoria (*n* = 7/81; 9%).

Trainee respondents were evenly distributed across the 5 years of training; four respondents indicated they were beyond their fifth year of training.

Over half of trainee respondents (*n* = 41/76; 54%) held another degree besides their medical degree, with the most common being a Bachelor of Science (*n* = 19), Bachelor of Medical Science (*n* = 6), Bachelor of Applied Science (*n* = 1) and Bachelor of Pharmacy (*n* = 1).

### 3.3 Trainee Perceptions of Radiation Oncology as a Career

Most trainee respondents (*n* = 67/75; 89%) were satisfied with radiation oncology as a career, and 83% (*n* = 62/75) satisfied with their training network. 16 per cent (*n* = 12/75) of trainee respondents would have reconsidered joining the specialty if they were aware of a perceived oversupply.

The top factors (in descending order) influencing career choice were interest in oncology patients, lifestyle after training, work hours and lifestyle during training. Trainees could have selected more than one factor.

### 3.4 Trainee Duties and Protected Time

Most trainees worked between 36 and 45 h (*n* = 31/76, 40%) and between 26 and 35 h (*n* = 21/76, 28%). 7% (*n* = 5/76) reported working over 55 h per week. Most respondents (*n* = 44/76, 58%) were on call less than or equal to 5 h per week; however, 11% (*n* = 8/76) reported being on call for over 20 h per week.

Trainees had a median of 2 h (range 0– ≥ 4) per week of protected time for teaching, but a median of 0 h (range 0– ≥ 4) for research and other activities.

### 3.5 Planning and Part‐Time Training

Most trainee respondents (*n* = 62/76; 82%) reported spending five or fewer hours on planning each week, with 70% (*n* = 53/76) spending less than 5 h per week contouring. A small number (*n* = 8/75; 11%) were working or planning to work part‐time for at least 12 months. 52 per cent (*n* = 39/75) reported a desire to work part‐time, with females (*n* = 23/33; 70%) more likely to do so than males (*n* = 16/42; 38%).

### 3.6 Challenges Associated With Radiation Oncology Training

The greatest sources of stress perceived by trainees were training demands (*n* = 70/81, 86%), balancing responsibilities (*n* = 53/81; 65%), job demands (*n* = 44/81; 54%) and job prospects (*n* = 41/81; 51%). Respondents could have selected more than one option, and percentages are therefore taken as a proportion of the full trainee cohort. Most trainees found a mentor to be useful (*n* = 62/81; 77%).

### 3.7 Impact of COVID‐19 on Training

Most trainees (*n* = 55/75; 73%) responded that COVID‐19 had a negative impact on their training. A small proportion of respondents (*n* = 17/75; 20%) felt the pandemic had influenced their decision regarding their future career, citing concerns such as work location and work–life balance. 79% (*n* = 59/75) of respondents indicated that COVID‐19 did not delay their exams; a further 92% (*n* = 70/76) reported that COVID‐19 had no influence on taking a fellowship position.

### 3.8 Trainees' Future Plans

Almost all trainee respondents (*n* = 71/75; 95%) intended to continue their career in radiation oncology, with 47% (*n* = 35/75) wanting to do a fellowship after training. The main reasons for pursuing a fellowship were to gain special skills and expertise (*n* = 34/35; 97%), to ease the transition into a consultant position (*n* = 25/35; 71%) and to be more competitive in the job market (*n* = 22/35; 63%).

When asked about their future practice, the majority of respondents (*n* = 58/75; 77%) showed interest in teaching and becoming a Director of Training (DoT) and/or an examiner, (*n* = 43/75, 57%) wished to participate in college committee work, (*n* = 40/75; 53%) are interested in clinical trials, (*n* = 15/75; 20%) wish to complete a higher degree during training and (*n* = 7/75; 9%) were already enrolled in a higher degree programme at the time of the survey. Respondents reported an interest in an academic career; predominantly, this was due to an interest in teaching (*n* = 46/75; 61%), clinical research (*n* = 23/75; 31%), a wish to practise in a large teaching centre (*n* = 22/75; 29%) and an interest in future leadership or administrative opportunities (*n* = 27/75; 36%). Those not interested in future academic work cited primary interest in patient care, politics/bureaucracy and a lack of interest in research as the main reasons.

Of trainee respondents, 89% (*n* = 67/75) were not in bonded medical places, and 69% (*n* = 52/75) indicated that they wanted a job where a subspecialty clinical interest could be pursued.

The 42 trainees (*n* = 42/75; 56%) who intended to work part‐time post‐training plan to work 0.5 to 0.6 FTE (*n* = 11/42; 26%), 0.7 to 0.9 FTE (*n* = 23/42; 55%) or were unsure (*n* = 8/42; 19%). Family commitments (*n* = 32/75; 42%), lifestyle (*n* = 28/75; 37%) and parental leave (*n* = 15/75; 20%) were cited as the main reasons for preferring part‐time employment.

There was a preference to work in an urban department by *n* = 33/75 trainee respondents (44%), with 32% (*n* = 24/75) undecided; 16% (*n* = 12/75) intend to do a combination of urban and rural practice and 8% (*n* = 6/75) intend to work in a rural department. These results mirror the 2018 census. Most trainees expressed an intent to work a mixture of public and private practice (*n* = 46/75, 61%) with 28 (37%) wanting to work in the public sector only. Only one respondent reported an exclusive interest in private practice.

### 3.9 Trainee Future Concerns

Job availability was the major concern for 77% (*n* = 58/75) of trainee respondents, followed by fellowship opportunities (*n* = 29/75; 39%).

### 3.10 Comments by Respondents

All respondents were given the opportunity to add further comments. There were 42 responses with very diverse answers. There were eight comments on increasing workload, five suggestions/criticisms of the census, four pieces of feedback for the college, three comments on the impact from Covid, two comments on the gender pay gap, two comments on increasing volume and contouring, and six respondents praised the census and the college.

## 4 Discussion

This is the seventh Faculty of Radiation Oncology workforce census and the fifth one this century. A separate New Zealand analysis will follow this census as in 2015 and 2019 [8, 9]. There continues to be an upward trend in the number of radiation oncologists in Australia, New Zealand (ANZ) and Singapore (*n* = 591) compared to previous censuses (*n* = 514 in 2018; 439 in 2014). Despite this, the response rate was lower (52%) compared with previous years (67.7% in 2018; 76.1% in 2014). The higher number of radiation oncologists correlates with an increased number of linacs over the past decade (*n* = 145 in 2011; *n* = 233 in 2021) [10].

COVID‐19 had a significant impact on the provision of cancer services across the world. Several guidelines [11] recommended the use of hypofractionated radiotherapy schedules to reduce patient exposure and optimise use of limited resources. Most respondents in this census (*n* = 202/210; 96%) indicated that COVID‐19 had little or no influence on the use of hypofractionation (Table 4). This is in contrast to trends in other countries such as the UK, where there was a rapid adoption of shorter schedules with breast (60% 5‐fraction) and palliative (50% single fraction) treatments, which coincided with the publication of trials such as FAST‐Forward in May 2020 [12] supporting the use of ultra‐hypofractionation for adjuvant breast cancer radiotherapy [13]. The majority of those surveyed (*n* = 112/210; 53%) expected to increase their use of hypofractionation in the future, regardless of the impact of COVID‐19, which is in keeping with the growing evidence base supporting the use of hypofractionation and stereotactic techniques. Patient wait times were mostly well managed by departments, with only 20% (*n* = 46/233) of respondents noting an increase in treatment waiting times during the pandemic.

The census revealed no Aboriginal/Torres Strait or Maori practitioners. There has been a strong desire to attract indigenous doctors into the RANZCR training programme; however, the impact has not yet been seen, suggesting that more needs to be done given the disparity in health outcomes between indigenous and non‐indigenous Australians. The RANZCR board has established the Maori, Aboriginal and Torres Strait Islander Executive Committee (MATEC) which has been incorporated into the College's strategic plan to address this issue [14]. The percentage of Asian representation amongst respondents has remained stable compared to the 2018 census (21%). The proportion of female radiation oncologists in the workforce remains similar at 42%.

Radiation oncologists who practise brachytherapy remain in the minority and almost unchanged compared to 2018 (*n* = 63). Reassuringly, the vast majority who do practise brachytherapy intend to continue in this area. The latest facilities survey report 2010–2020 has highlighted that the use of brachytherapy for gynaecological and prostate cancers has fallen, which is a significant concern [10]. A brachytherapy working group has been created within the College with the remit to review current workforce practices (and gaps) and look for opportunities to strengthen services in the future.

With the addition of a new question regarding the field of theranostics, only 5 respondents (1.8%) answered that they currently practise in this area, with the majority treating thyroid cancers and all working in the public sector. Most received training in this area as part of their registrar training. There has been a position statement produced by the Australasian Association of Nuclear Medicine Specialists (AANMS) on the practice of theranostics in Australia and RANZCR has now formed a Theranostics Working Group to provide further guidance on this.

Respondents were asked to list their places of employment and the type of workplace that it was (Public, private or public/private). Notably, the number of respondents who worked exclusively in the private sector across their different workplaces has slightly increased when compared to previous years (*n* = 32/243; 13%), versus 11% in 2018) which is likely related to the growth of new private practices in Australia over the past decade. In 2021, 60% of radiation therapy facilities in Australia were privately operated compared with 35% in 2011. In terms of distribution of linacs, in 2021, 101 linacs were in the private sector and 132 in public. In addition to the 13% working exclusively in private, 40% (*n* = 97/243) work across a mix of public and private, which if combined, shows that 53% of respondents work in private practice for at least some of their week, which reflects the growth in private practice.

Of concern, radiation oncologists in New Zealand and WA have reported the highest working hours per week (50.4 and 52.5 h respectively) and highlights the need to address workforce shortages in these areas. The upper range of hours worked in some states (upwards of 70–80 h per week) raises potential concerns around burnout, personal wellbeing and clinical performance amongst our respondents. Likewise, the number of new cases seen by each respondent per year (Figure 2) showed that a significant percentage (*n* = 45/210; 21%) see more than 300 cases with a select few consulting on up to 700 per year. Further breakdown regarding the comparison between caseload in public and private practice was not recorded. These figures by no means form any benchmark by RANZCR on recommended caseload and have only been reported as stated.

Reassuringly, all five censuses this century have revealed low unemployment amongst radiation oncologists; 1.4% in this census (comparable with 1.3% in 2018; 1.5% in 2014). This trend appears to be consistent with workforce planning projections in Canada [15] and the United States until 2030–2040 [16]. These results are very pleasing given that there was significant pressure faced by RANZCR from its membership in the past decade to reduce trainee numbers due to concerns about job availability. The high employment rates for new fellows suggest that the trainee numbers were not in excess during this period.

A recent article by Yap et al. [17] highlighted the gender pay gap that exists in radiation oncology. This workforce survey showed that both male and female radiation oncologists worked similar hours (median 43 h (M) vs. 42 h (F)) although male respondents did see more new patients per year compared with female respondents (281 vs. 211 respectively). There were also more males than females having either incentive‐based or mixed income (53% vs 44%; Table 5) so this may partly contribute to the gender pay gap seen.

In comparison to the previous census, there was a slightly higher number of radiation oncologists planning to reduce their hours in the next 3 years (26% vs 22%); however, a greater number are intending to retire within the next 6–15 years (42% vs. 35%). Although not directly collected in this census, from RANZCR records we know that the average age of our members is gradually increasing (49 years in 2022 compared with 47 in 2018). This is a concern, especially in New Zealand where a significant workforce shortfall is forecasted in the coming decade [14]. Future succession planning needs to be considered at both a departmental and a national level.

Other responses relating to the hours spent per week on new patients, follow up, and treatment review, contouring, research, teaching and management remained similar to previous censuses.

### 4.1 Trainees

Trainee numbers in this census have increased (*n* = 167) compared with 2018 (*n* = 140). Despite the high rates of employment following fellowship, job prospects remain the highest concern for the future although the vast majority aim to continue their training (95%). Despite the high levels of concern about job opportunities in the context of an increase in trainee numbers, there are several factors that indicate ongoing strong job growth and employment. These include an ageing RO workforce, long‐term stable unemployment (< 2%), the finding that 56% of trainees do not intend to work full time, and 26% of radiation oncologists are planning to reduce their hours in the next 3 years. Along with expanded linac numbers, RANZCR members are able to reassure current and prospective trainees about job prospects. This reassurance should be part of the role of clinical supervisors as it is clear this is a concern to trainees.

The majority of trainees felt that COVID‐19 had a negative impact on training (72%) which is significantly higher compared with oncology trainee experiences in Canada (46%) [18] and Europe (43%) [19]. Up to 21% reported that COVID‐19 resulted in a delay in taking exams and influenced their decision regarding future career choices. This may have in part contributed to the observed decline in the number of full‐time equivalent radiation oncologists between 2019 and 2021, as shown in the Facilities Survey [10]. The vast majority were not deployed to work outside of their specialty field during the pandemic and did not have any reduction in work hours or salary.

Most (75%) had the same workload, which they managed either in the workplace or while working from home. Balancing work and family responsibilities and job demands was the two areas that caused the most stress to trainees. As a comparison, a longitudinal survey of oncology trainees conducted by ESMO [19] found almost half had increased working hours, with 45% finding inadequate time for personal or family life. From the same survey, a quarter had considered changing their future career, and more than half felt burnt out. Identifying burn‐out in oneself and others is one of the roles in the CANMEDS framework and has been highlighted in a previous trainee survey by Leung et al. [20] as being highly prevalent (almost 50%) and deserves closer attention at a College level.

Most trainees are satisfied with their training network (83%) which is almost twice that of their medical oncology counterparts (42%) in Australia from a recent survey [21]. 16 percent of trainees would have reconsidered the specialty had they been aware of a perceived oversupply which is largely unchanged from the previous census.

Of concern, 70% of trainees spend less than 5 h per week contouring, which is slightly higher than in 2018 (64%). A trainee survey by Leung et al. in 2019 [22] revealed that the majority of respondents had insufficient time for contouring and over half had no allocated time. With the recent changes in the training programme and the implementation of work‐based assessments and contouring/plan review forming an integral part of this, this is likely to improve in the future, and it will be interesting to evaluate the trend in future censuses.

Nine percent of trainees reported no protected teaching time, which is an improvement on previous years (15% in 2018 and 2014); however, those receiving 4 or more hours per week of protected time fell slightly to 18% (23% in 2018). This is a concern given that trainees at college‐accredited training sites should be provided with at least 4 h of protected time per week and may reflect the anonymity bias of this census.

There has been a significant reduction in the number of trainees wanting to pursue a fellowship after training (47% versus 70% in the previous census). This may reflect changing clinical practice; previously subspecialised techniques such as SBRT/SRS/SRT now form part of the core curriculum for trainees. The reasons for planning to complete a fellowship remained the same: to gain expertise, ease transition to consultant, and to be more competitive in the job market. Of interest, 32% of trainees remain undecided on their preferred future workplace location (metro versus regional) which highlights the potential to implement strategies to attract more new fellows to regional practice.

## 5 Conclusion

The 2022 RANZCR FRO workforce census has continued the accumulation of data from previous censuses. Despite a global pandemic, the impact of COVID‐19 on local practices and workloads was not as significant as seen overseas. There continues to be an increasing trend of radiation oncologists working in the private sector, which correlates with the significant growth in private centres over the past decade. There remains a number of similarities compared with the previous census, including hours spent per week on new patients, follow up, and treatment review, contouring, research, teaching and management. Regarding trainees, there has been a decrease in the number of new fellows seeking to complete further fellowships, which may be a reflection of the availability of consultant positions. The lack of indigenous representation within our profession continues to be an area that needs further attention.

## Author Contributions

**Hon Trinh:** writing – original draft, writing – review and editing, methodology, formal analysis. **Gerard Adams:** methodology, writing – review and editing. **rap chee:** methodology, writing – review and editing. **Tuan Ha:** methodology, writing – review and editing. **Marcel Knesl:** writing – review and editing. **Jack Mitchell:** writing – review and editing. **Sakshi Nagpal:** formal analysis, data curation. **Edward Sia:** writing – review and editing. **Nathan Stevens:** writing – review and editing, formal analysis, data curation. **Daniel Xing:** methodology, writing – review and editing.

## Disclosure

This census was conducted as a voluntary survey of medical practitioners which involved no risk to themselves or patients. All results published are non‐identifiable.

## Conflicts of Interest

No authors have any conflicts of interest to disclose.

## Supporting information


**Data S1:** 2022 Radiation Oncology workforce census.

## Data Availability

The data that support the findings of this study are available from the corresponding author upon reasonable request.
