# Supplementary material for: Faculty of Radiation Oncology 2022 Workforce Census
Source: J Med Imaging Radiat Oncol. 2025 Aug 7;69(6):687–95. doi: 10.1111/1754-9485.13883 (PMC12418052; doi:10.1111/1754-9485.13883)
Supplement: Supplementary file 1 — Data S1: 2022 Radiation Oncology workforce census. [file ARA-69-687-s001.pdf]

The Royal Australian and New Zealand College of Radiologists®
The Faculty of Radiation Oncology

2022 Radiation Oncology Workforce Census

Introduction

Dear Colleague,

You are invited to participate in the 2022 RANZCR Radiation Oncology Workforce Census. All responses will be treated with the strictest confidence and will only be viewed by the Analyst - Economics and Analytics.

As you know, radiation oncology faces many challenges – clinical, professional and economic. The results of this census will be used to collaborate with governments for better resourcing for radiation oncology (e.g., caseload recommendations; workforce planning) and internally to guide development of services to you as Fellows and Members of the College.

All completed responses received by **Wednesday 31 August 2022** will go in the draw to win an **iPad mini 4**.

Thank you in advance for your time.

Best regards,

Economics & Workforce Committee members:

- Dr Gerry Adams (Chair)
- Dr Keen-Hun Tai (Dean)
- Dr Giuseppe Sasso
- A/Prof Jonathan Ramsay
- Dr Hon Trinh
- A/Prof Raphael Chee
- Dr Craig Wilson
- Dr Katherine Meng

The Royal Australian and New Zealand College of Radiologists®
The Faculty of Radiation Oncology

2022 Radiation Oncology Workforce Census

Demographics: Ethnicity and category

1. Please identify the ethnicity that you MOST identify with

(if other, please specify)

2. Please indicate the category that best describes you as at 30 June 2022:

- FRANZCR currently working in Australia, including some clinical work Q3
- FRANZCR currently working in New Zealand, including some clinical work Q3
- FRANZCR currently working in Australia, but in non-clinical areas only Q3
- FRANZCR currently working in New Zealand, but in non-clinical areas only Q3
- FRANZCR currently working outside Australia/New Zealand Q207
- FRANZCR currently on leave Q4
- FRANZCR currently unemployed and actively seeking work Q4
- FRANZCR currently unemployed and not actively seeking work Q4
- FRANZCR currently retired Q160
- IMG working in an Area of Need position as a consultant in Australia Q4
- IMG going through the specialist recognition pathway Q221?
- IMG obtaining short term training Q221? Q208
- IMG working in New Zealand within a Vocational Scope of Practice Q3
- Trainee in the RANZCR training program, currently working as a registrar in a RANZCR-accredited training position in Australia Q161
- Trainee in the RANZCR training program, currently working as a registrar in a RANZCR-accredited training position in New Zealand Q161
- Trainee in the RANZCR training program, currently working in a 5th year Fellowship position Q161
- Trainee in Australian RANZCR training program position, currently on a break, not working as a registrar or working outside Australia End
- Trainee in New Zealand RANZCR training program position, currently on a break, not working as a registrar or working outside New, Zealand End
- Former trainee in the RANZCR training program, currently working in a non-accredited position Q221? Q208

2022 Radiation Oncology Workforce Census

Demographics: Employment

3. Please select the category that best fits your current employment.

- ◯ Specialist/consultant radiation oncologist in Australia
- ◯ Specialist/consultant radiation oncologist in New Zealand
- ◯ Specialist/consultant radiation oncologist overseas
- ◯ Locum radiation oncologist in Australia
- ◯ Locum radiation oncologist in New Zealand
- ◯ Locum radiation oncologist overseas
- ◯ Other (please specify)
- ◯ Undertaking Fellowship position in Australia
- ◯ Undertaking Fellowship position in New Zealand
- ◯ Undertaking Fellowship position overseas
- ◯ Non-clinical position in Australia
- ◯ Non-clinical position in New Zealand
- ◯ Non-clinical position overseas

The Royal Australian and New Zealand College of Radiologists®
The Faculty of Radiation Oncology

2022 Radiation Oncology Workforce Census

Demographics: Medical qualifications

4. In which year did you graduate from medical school?

Please enter in YYYY format (e.g. 2018): ____________

5. Do you hold a Graduate research higher degree (RHD)? (PLEASE NOTE: Primary medical qualifications and FRANZCR are not considered as higher degrees)

- ○ Yes
- ○ No

2022 Radiation Oncology Workforce Census

Demographics: Higher degree

If you have a Graduate research higher degree (RHD) please specify the following information:

PLEASE NOTE: Primary medical qualifications and FRANZCR are not considered as higher degrees

6. Degree type:

7. Degree name:

8. Institution name:

9. Year awarded:

Please enter in YYYY format (e.g. 2018):

2022 Radiation Oncology Workforce Census

Demographics: Special interests

10. Do you hold special interests in any of the following areas? (Please select all that apply)

- [ ] Brachytherapy
- [ ] Breast
- [ ] Endocrine
- [ ] Gastrointestinal
- [ ] Gynae-oncology
- [ ] Head and neck
- [ ] Lung
- [ ] Lymphoma
- [ ] Neurological cancers
- [ ] Paediatrics
- [ ] Palliative
- [ ] Sarcoma
- [ ] Skin
- [ ] Stereotactic
- [ ] Theranostics
- [ ] Urological
- [ ] Other (please specify)

The Royal Australian and New Zealand College of Radiologists®
The Faculty of Radiation Oncology

2022 Radiation Oncology Workforce Census

Brachytherapy

11. What percentage of your time is spent doing brachytherapy work?

0 100

12. What percentage of your time do you spend doing the following work? (Enter numbers between 0-100% only)

Please note the sum of the percentage answers given Q12 should equal the total percentage of your time spent doing brachytherapy work given in Q11.

Gynae-oncology

Uro-oncology – LDR

Uro-oncology – HDR

Other

13. If other in Q12, please specify.

14. Do you plan on continuing to perform brachytherapy?

- Yes
- No
- I do not perform brachytherapy

If no, please specify the reason of not continuing brachytherapy.

2022 Radiation Oncology Workforce Census

Theranostics

15. Do you currently practice theranostics?

- ◯ Yes Q16
- ◯ No Q23

If you are not currently practicing theranostics, are you interested in doing theranostics?

2022 Radiation Oncology Workforce Census

Theranostics

16. What percentage of your time is spent on theranostics?

0% 100%

17. For how long have you been practicing theranostics?

18. How long do you plan to continue practicing theranostics?

0 years 20+ years

19. How many patients do you treat per year?

20. Please give the percentage of patients from each tumour group that makes up your practice (the total should add up to 100%).

Thyroid

Neuroendocrine

Prostate

Other (please specify the % and tumour group)

21. Is your theranostics work

- Fully at a public facility
- Fully at a private facility
- A mixture of both

22. What training/accreditation have you had within the field

The Royal Australian and New Zealand College of Radiologists®
The Faculty of Radiation Oncology

2022 Radiation Oncology Workforce Census

Leadership positions

23. Do you currently hold any leadership positions? (e.g. College Committee, Director of Department, Director of Training and other leadership positions)

If so, please specify the name of the position and how long you have held the position.

Leadership position 1

Leadership position 2

Leadership position 3

Leadership position 4

Leadership position 5

Leadership position 6

Leadership position 7

Leadership position 8

Leadership position 9

Leadership position 10

2022 Radiation Oncology Workforce Census

Practice location and work hours: Work status

24. Do you identify as:

- ◯ Working in a clinical position Q25
- ◯ Working in a non-clinical position Q207
- ◯ On leave from work/training Q159
- ◯ Unemployed Q207
- ◯ Other (please specify) Q213

2022 Radiation Oncology Workforce Census

Practice location and work hours: Number of practices

25. Please indicate the number of Hospital Departments and/or Practices that you work at, including any outreach clinics.

- ◯ 1
- ◯ 2
- ◯ 3
- ◯ 4
- ◯ 5
- ◯ 6
- ◯ 7
- ◯ 8
- ◯ 9
- ◯ 10

2022 Radiation Oncology Workforce Census

Practice location and work hours: Workplace 1

26. Name of Department/Practice

27. Name of Hospital/Corporation

28. Suburb:

29. Postcode:

30. State/Territory/Country

31. Is this workplace:

- Public
- Private
- Public/private

32. Is your source of funding for this workplace:

- Public
- Private
- Public/private

33. What formal Full Time Equivalent (FTE) are you contracted to work for this workplace? (i.e. 0.1 FTE = 4hrs; 0.4 FTE = 2 days; 1.0 FTE = 5 day week.)

- 0.1
- 0.2
- 0.3
- 0.4
- 0.5
- 0.6
- 0.7
- 0.8
- 0.9
- 1.0

34. In a typical working week, how many hours do you work:

At this site

For this site but remotely (i.e. at home or at another workplace)

35. Of the hours worked in a typical working week, please estimate how many hours are spent on clinical work.

36. Please indicate here if you DO NOT work at any more Departments or Practices:

- ◯ I do not work at any more Departments/Practices

The Royal Australian and New Zealand College of Radiologists®
The Faculty of Radiation Oncology

2022 Radiation Oncology Workforce Census

Practice location and work hours: Workplace 2

37. Name of Department/Practice

38. Name of Hospital/Corporation

39. Suburb:

40. Postcode:

41. State/Territory/Country

42. Is this workplace:

- ◯ Public
- ◯ Private
- ◯ Public/private

43. Is your source of funding for this workplace:

- ◯ Public
- ◯ Private
- ◯ Public/private

44. What formal Full Time Equivalent (FTE) are you contracted to work for this workplace? (i.e. 0.1 FTE = 4hrs; 0.4 FTE = 2 days; 1.0 FTE = 5 day week.)

- ◯ 0.1
- ◯ 0.2
- ◯ 0.3
- ◯ 0.4
- ◯ 0.5
- ◯ 0.6
- ◯ 0.7
- ◯ 0.8
- ◯ 0.9
- ◯ 1.0

45. In a typical working week, how many hours do you work:

At this site [ ]

For this site but remotely (i.e. at home or at another workplace) [ ]

46. Of the hours worked in a typical working week, please estimate how many hours are spent on clinical work.

[ ]

47. Please indicate here if you DO NOT work at any more Departments or Practices:

- ○ I do not work at any more Departments/Practices

The Royal Australian and New Zealand College of Radiologists®
The Faculty of Radiation Oncology

2022 Radiation Oncology Workforce Census

Practice location and work hours: Workplace 3

48. Name of Department/Practice

49. Name of Hospital/Corporation

50. Suburb:

51. Postcode:

52. State/Territory/Country

53. Is this workplace:

- Public
- Private
- Public/private

54. Is your source of funding for this workplace:

- Public
- Private
- Public/private

55. What formal Full Time Equivalent (FTE) are you contracted to work for this workplace? (i.e. 0.1 FTE = 4hrs; 0.4 FTE = 2 days; 1.0 FTE = 5 day week.)

- 0.1
- 0.2
- 0.3
- 0.4
- 0.5
- 0.6
- 0.7
- 0.8
- 0.9
- 1.0

56. In a typical working week, how many hours do you work:

At this site

For this site but remotely (i.e. at home or at another workplace)

57. Of the hours worked in a typical working week, please estimate how many hours are spent on clinical work.

58. Please indicate here if you DO NOT work at any more Departments or Practices:

- ◯ I do not work at any more Departments/Practices

2022 Radiation Oncology Workforce Census

Practice location and work hours: Workplace 4

59. Name of Department/Practice

60. Name of Hospital/Corporation

61. Suburb:

62. Postcode:

63. State/Territory/Country

64. Is this workplace:

- Public
- Private
- Public/private

65. Is your source of funding for this workplace:

- Public
- Private
- Public/private

66. What formal Full Time Equivalent (FTE) are you contracted to work for this workplace? (i.e. 0.1 FTE = 4hrs; 0.4 FTE = 2 days; 1.0 FTE = 5 day week.)

- 0.1
- 0.2
- 0.3
- 0.4
- 0.5
- 0.6
- 0.7
- 0.8
- 0.9
- 1.0

67. In a typical working week, how many hours do you work:

At this site [ ]

For this site but remotely (i.e. at home or at another workplace) [ ]

68. Of the hours worked in a typical working week, please estimate how many hours are spent on clinical work.

[ ]

69. Please indicate here if you DO NOT work at any more Departments or Practices:

- ◯ I do not work at any more Departments/Practices

The Royal Australian and New Zealand College of Radiologists®
The Faculty of Radiation Oncology

2022 Radiation Oncology Workforce Census

Practice location and work hours: Workplace 5

70. Name of Department/Practice

71. Name of Hospital/Corporation

72. Suburb:

73. Postcode:

74. State/Territory/Country

75. Is this workplace:

- ◯ Public
- ◯ Private
- ◯ Public/private

76. Is your source of funding for this workplace:

- ◯ Public
- ◯ Private
- ◯ Public/private

77. What formal Full Time Equivalent (FTE) are you contracted to work for this workplace? (i.e. 0.1 FTE = 4hrs; 0.4 FTE = 2 days; 1.0 FTE = 5 day week.)

- ◯ 0.1
- ◯ 0.2
- ◯ 0.3
- ◯ 0.4
- ◯ 0.5
- ◯ 0.6
- ◯ 0.7
- ◯ 0.8
- ◯ 0.9
- ◯ 1.0

78. In a typical working week, how many hours do you work:

At this site

For this site but remotely (ie at home or at another workplace)

79. Of the hours worked in a typical working week, please estimate how many hours are spent on clinical work.

80. Please indicate here if you DO NOT work at any more Departments or Practices:

- ◯ I do not work at any more Departments/Practices

2022 Radiation Oncology Workforce Census

Practice location and work hours: Workplace 6

81. Name of Department/Practice

82. Name of Hospital/Corporation

83. Suburb:

84. Postcode:

85. State/Territory/Country

86. Is this workplace:

- Public
- Private
- Public/private

87. Is your source of funding for this workplace:

- Public
- Private
- Public/private

88. What formal Full Time Equivalent (FTE) are you contracted to work for this workplace? (i.e. 0.1 FTE = 4hrs; 0.4 FTE = 2 days; 1.0 FTE = 5 day week.)

- 0.1
- 0.2
- 0.3
- 0.4
- 0.5
- 0.6
- 0.7
- 0.8
- 0.9
- 1.0

89. In a typical working week, how many hours do you work:

At this site [ ]

For this site but remotely (ie at home or at another workplace) [ ]

90. Of the hours worked in a typical working week, please estimate how many hours are spent on clinical work.

[ ]

91. Please indicate here if you DO NOT work at any more Departments or Practices:

- ◯ I do not work at any more Departments/Practices

2022 Radiation Oncology Workforce Census

Practice location and work hours: Workplace 7

92. Name of Department/Practice

93. Name of Hospital/Corporation

94. Suburb:

95. Postcode:

96. State/Territory/Country

97. Is this workplace:

- ◯ Public
- ◯ Private
- ◯ Public/private

98. Is your source of funding for this workplace:

- ◯ Public
- ◯ Private
- ◯ Public/private

99. What formal Full Time Equivalent (FTE) are you contracted to work for this workplace? (i.e. 0.1 FTE = 4hrs; 0.4 FTE = 2 days; 1.0 FTE = 5 day week.)

- ◯ 0.1
- ◯ 0.2
- ◯ 0.3
- ◯ 0.4
- ◯ 0.5
- ◯ 0.6
- ◯ 0.7
- ◯ 0.8
- ◯ 0.9
- ◯ 1.0

100. In a typical working week, how many hours do you work:

At this site [ ]

For this site but remotely (i.e. at home or at another workplace) [ ]

101. Of the hours worked in a typical working week, please estimate how many hours are spent on clinical work.

[ ]

102. Please indicate here if you DO NOT work at any more Departments or Practices:

- ○ I do not work at any more Departments/Practices

2022 Radiation Oncology Workforce Census

Practice location and work hours: Workplace 8

103. Name of Department/Practice

104. Name of Hospital/Corporation

105. Suburb:

106. Postcode:

107. State/Territory/Country

108. Is this workplace:

- ◯ Public
- ◯ Private
- ◯ Public/private

109. Is your source of funding for this workplace:

- ◯ Public
- ◯ Private
- ◯ Public/private

110. What formal Full Time Equivalent (FTE) are you contracted to work for this workplace? (i.e. 0.1 FTE = 4hrs; 0.4 FTE = 2 days; 1.0 FTE = 5 day week.)

- ◯ 0.1
- ◯ 0.2
- ◯ 0.3
- ◯ 0.4
- ◯ 0.5
- ◯ 0.6
- ◯ 0.7
- ◯ 0.8
- ◯ 0.9
- ◯ 1.0

111. In a typical working week, how many hours do you work:

At this site [ ]

For this site but remotely (i.e. at home or at another workplace) [ ]

112. Of the hours worked in a typical working week, please estimate how many hours are spent on clinical work.

[ ]

113. Please indicate here if you DO NOT work at any more Departments or Practices:

- ◯ I do not work at any more Departments/Practices

The Royal Australian and New Zealand College of Radiologists®
The Faculty of Radiation Oncology

2022 Radiation Oncology Workforce Census

Practice location and work hours: Workplace 9

114. Name of Department/Practice

115. Name of Hospital/Corporation

116. Suburb:

117. Postcode:

118. State/Territory/Country

119. Is this workplace:

- ◯ Public
- ◯ Private
- ◯ Public/private

120. Is your source of funding for this workplace:

- ◯ Public
- ◯ Private
- ◯ Public/private

121. What formal Full Time Equivalent (FTE) are you contracted to work for this workplace? (i.e. 0.1 FTE = 4hrs; 0.4 FTE = 2 days; 1.0 FTE = 5 day week.)

- ◯ 0.1
- ◯ 0.2
- ◯ 0.3
- ◯ 0.4
- ◯ 0.5
- ◯ 0.6
- ◯ 0.7
- ◯ 0.8
- ◯ 0.9
- ◯ 1.0

122. In a typical working week, how many hours do you work:

At this site [ ]

For this site but remotely (i.e. at home or at another workplace) [ ]

123. Of the hours worked in a typical working week, please estimate how many hours are spent on clinical work.

[ ]

124. Please indicate here if you DO NOT work at any more Departments or Practices:

○ I do not work at any more Departments/Practices

The Royal Australian and New Zealand College of Radiologists®
The Faculty of Radiation Oncology

2022 Radiation Oncology Workforce Census

Practice location and work hours: Workplace 10

125. Name of Department/Practice

126. Name of Hospital/Corporation

127. Suburb:

128. Postcode:

129. State/Territory/Country

130. Is this workplace:

- ◯ Public
- ◯ Private
- ◯ Public/private

131. Is your source of funding for this workplace:

- ◯ Public
- ◯ Private
- ◯ Public/private

132. What formal Full Time Equivalent (FTE) are you contracted to work for this workplace? (i.e. 0.1 FTE = 4hrs; 0.4 FTE = 2 days; 1.0 FTE = 5 day week.)

- ◯ 0.1
- ◯ 0.2
- ◯ 0.3
- ◯ 0.4
- ◯ 0.5
- ◯ 0.6
- ◯ 0.7
- ◯ 0.8
- ◯ 0.9
- ◯ 1.0

133. In a typical working week, how many hours do you work:

At this site

For this site but remotely (i.e. at home or at another workplace)

134. Of the hours worked in a typical working week, please estimate how many hours are spent on clinical work.

2022 Radiation Oncology Workforce Census

Work hours

For the following questions a new case is defined as "a patient seen either face to face or by a recognised telehealth mechanism that has not been previously seen for the same diagnosis. It does not include re-treatments of patients or those discussed at multidisciplinary meetings who are not being referred for you to see or consult".

PLEASE NOTE: For decimal figures, please estimate to the nearest whole number.

135. In a typical working week, approximately how many hours do you spend on MDTs:

136. In a typical working week, approximately how many hours do you spend on:

New cases (hours per week)

Follow up cases (hours per week)

'On treatment' reviews (hours per week)

Simulation (hours per week)

Dosimetry (hours per week)

Contouring (hours per week)

137. On average, how long do you allow for each (includes documentation and dictation):

New case (minutes per case)

Follow up case (minutes per case)

'On treatment' review case (minutes per case)

138. In a typical working week, approximately how many hours do you spend on the following non-clinical activities (non-mdm):

Supervision of registrars (hours per week)

Research and trials (hours per week)

Department/Practice management (hours per week)

Academic teaching (hours per week)

Jurisdiction/Hospital/quality committees (hours per week)

Other (hours per week)

139. If other, please specify

140. In a typical working week how many hours do you spend working

Physically on site

Remotely

2022 Radiation Oncology Workforce Census

Patterns of Practice: FTE and patients

141. What is your total Full Time Equivalent (FTE) status? (i.e. 0.1 FTE = 4hrs; 0.4 FTE = 2 days; 1.0 FTE = 5 day week.)

- ◯ 0.1
- ◯ 0.2
- ◯ 0.3
- ◯ 0.4
- ◯ 0.5
- ◯ 0.6
- ◯ 0.7
- ◯ 0.8
- ◯ 0.9
- ◯ 1.0
- ◯ 1.0+ (please specify)

142. Across all your workplaces, how many NEW patients would you consult on in a year?

143. Do you manage inpatients under your own bedcard?

- ◯ No
- ◯ Yes (please specify how many per year)

144. Do you think it is worthwhile to manage inpatients under our own bedcards?

- ◯ Yes
- ◯ No

2022 Radiation Oncology Workforce Census

Patterns of Practice: Specialisation

145. Do you consider yourself a 'generalist' or a 'specialist' radiation oncologist? (e.g., a Head & Neck specialist)

- ◯ Generalist
- ◯ Specialist

If you selected Specialist, please specify specialist area

[ ]

146. Have you taken up hypofractionation for the following:

	No	Sometimes	Half the time	Most times	Always	N/A
Breast (15/16 fractions)	◯	◯	◯	◯	◯	◯
Breast (5 fractions)	◯	◯	◯	◯	◯	◯
GBM (elderly 15 fractions)	◯	◯	◯	◯	◯	◯
Preoperative rectum (5 fractions)	◯	◯	◯	◯	◯	◯
Prostate (20 fractions) – excluding ultra hypofractionation (eg 7 or less)	◯	◯	◯	◯	◯	◯
Single fraction for bone metastases – excluding SBRT	◯	◯	◯	◯	◯	◯
Curative intent lung cancer (excluding SBRT) (eg 20 fraction schedule)	◯	◯	◯	◯	◯	◯

147. Has your answer to the hypofractionation question been influenced by the impact of COVID-19?

- ◯ Not at all
- ◯ A little
- ◯ A lot

148. When the impact of COVID-19 finishes, with regard to hypofractionation do you expect your practice to:

- ◯ Revert back to pre COVID-19 levels
- ◯ Stay at COVID-19 impacted levels
- ◯ Continue to increase regardless of COVID-19 impact
- ◯ Other (please specify)

149. Do you practice SBRT? If so, please specify which sites.

- ◯ No
- ◯ Yes (please specify which sites)

150. Do you practice SRS/SRT? If so, please specify which sites.

- ◯ Yes
- ◯ No

If yes, please specify which sites.

2022 Radiation Oncology Workforce Census

Remuneration

151. What is your income type?

- ◯ Fixed
- ◯ Incentive based
- ◯ Mixed
- ◯ Other (please specify)

2022 Radiation Oncology Workforce Census

Plans

152. Do you intend to change your hours of work in the next three (3) years?

- ◯ No
- ◯ Yes, reduce hours
- ◯ Yes, increase hours

153. Do you intend to retire in the next:

- ◯ 0-5 years
- ◯ 6-10 years
- ◯ 11-15 years
- ◯ Not in the foreseeable future

The Royal Australian and New Zealand College of Radiologists®
The Faculty of Radiation Oncology

2022 Radiation Oncology Workforce Census

Challenges

154. Please indicate whether you consider the following options to be a great source of stress for you:

	Strongly disagree	Disagree	Neutral or unsure	Agree	Strongly agree
Balancing work and family responsibilities	◯	◯	◯	◯	◯
Job demands	◯	◯	◯	◯	◯
Training demands	◯	◯	◯	◯	◯
Spending time in remote centres	◯	◯	◯	◯	◯
Job prospects	◯	◯	◯	◯	◯
Other (please specify below)	◯	◯	◯	◯	◯

Other (please specify)

155. How strongly do you consider leaving the specialty?

Not at all	Slightly	Neutral or unsure	Strongly	Very strongly
◯	◯	◯	◯	◯

Please add any comments to qualify your answer

156. Over the past year, I have had enough time to pursue interests outside of work:

Strongly disagree	Disagree	Neutral or unsure	Agree	Strongly agree
◯	◯	◯	◯	◯

157. How satisfied are you with your work?

- ◯ Very satisfied
- ◯ Satisfied
- ◯ Neither satisfied nor dissatisfied
- ◯ Dissatisfied
- ◯ Very dissatisfied

158. Please rate your level of work-related stress on:

	Not stressed at all	A little stressed	Moderately stressed	Very stressed	Extremely stressed
An average work day	◯	◯	◯	◯	◯
A peak work day	◯	◯	◯	◯	◯

2022 Radiation Oncology Workforce Census

Not in workforce: On leave

159. If you are on leave from work/training please indicate when you intend to re-enter the workforce:

- ◯ 0-1 year
- ◯ >1-2 years
- ◯ >2-5 years
- ◯ Not sure/unknown

2022 Radiation Oncology Workforce Census

Not in workforce: Retired

160. If you have already retired please enter either your age at retirement OR the date/year in which you retired:

The Royal Australian and New Zealand College of Radiologists®
The Faculty of Radiation Oncology

2022 Radiation Oncology Workforce Census

Trainee: Demographics

161. What year of training are you in?

162. Do you have another degree besides your medical qualification?

- No
- Yes (Please specify)

163. Do you have another specialist qualification?

- No
- Yes (Please specify)

2022 Radiation Oncology Workforce Census

Trainee: Choice of career

164. Which factors influenced/are influencing your career choice: (Please select all that apply)

- [ ] Training program reputation
- [ ] Reputation of staff
- [ ] Previous attachment as student or junior doctor
- [ ] Interest in oncology patients
- [ ] Interest in physics
- [ ] Interest in radiobiology
- [ ] Work hours
- [ ] Use of technology
- [ ] Lifestyle during training
- [ ] Lifestyle after training
- [ ] Family considerations
- [ ] Earning potential
- [ ] Research opportunities
- [ ] Job availability
- [ ] Other (please specify)

2022 Radiation Oncology Workforce Census

Trainee: Registrar duties and service: Clinical

165. During the past 6 months, on average how many hours per week did you spend on Clinical Time? (i.e. Any time Trainees spend involved in patient care and includes clinical assessment, planning, treatment, treatment reviews, ward work, clinical procedures, and clerical/administrative duties relating to patient care.)

- ≤ 15 hours
- 16-25 hours
- 26-35 hours
- 36-45 hours
- 46-55 hours
- > 55 hours

166. During the past 6 months, on average how many hours per week did you spend on after hours on call?

- ≤ 5 hours
- 6-10 hours
- 11-15 hours
- 16-20 hours
- > 20 hours

167. During the past 6 months, on average how many hours per week did you spend on planning?

- ≤ 5 hours
- 6-10 hours
- 11-15 hours
- 16-20 hours
- > 20 hours

168. During the past 6 months, on average how many hours per week did you spend on contouring?

- ◯ ≤ 5 hours
- ◯ 6-10 hours
- ◯ 11-15 hours
- ◯ 16-20 hours
- ◯ > 20 hours

2022 Radiation Oncology Workforce Census

Trainee: Registrar duties and service: Non-clinical

For the following questions, non-clinical time refers to time spent on activities related to study and other educational activities. This may include formal and semi-formal tutorial sessions, and administrative duties un-related to patient care. It includes preparation of curriculum assignments and case reports.

169. During the past 6 months, on average how many hours per week did you spend on Non-Clinical Time after hours?

- ≤ 5 hours
- 6-10 hours
- 11-15 hours
- 16-20 hours
- > 20 hours

170. During the past 6 months, on average how many hours per week did you spend on non-clinical tasks and NOT related to training, such as administrative and clerical work?

- ≤ 5 hours
- 6-10 hours
- 11-15 hours
- 16-20 hours
- > 20 hours

2022 Radiation Oncology Workforce Census

Trainee: Registrar duties and service: Protected time

For the next three questions, 'protected time' refers to the time that trainees are not available for normal clinical duties and is therefore part of non-clinical time. This requires that their clinical responsibilities are covered for the period of the 'protected time' by their peers and/or senior colleagues. This time is intended to be used for formal teaching activities, research activities and for gaining practical experience in planning and treatment as stipulated by the curriculum.

171. During the past 6 months, on average how many hours 'protected time' per week did you have to spend on tutorials and teaching?

- 0 hours
- ≤ 1 hour
- 2 hours
- 3 hours
- 4 hours
- > 4 hours

172. During the past 6 months, on average how many hours 'protected time' per week did you have to spend on research activities?

- 0 hours
- ≤ 1 hour
- 2 hours
- 3 hours
- 4 hours
- > 4 hours

173. During the past 6 months, on average how many hours 'protected time' per week did you have to spend on other activities? (i.e. the time that trainees are not available for normal clinical duties and is therefore part of non-clinical time.)

- 0 hours
- ≤ 1 hour
- 2 hours
- 3 hours
- 4 hours
- > 4 hours

174. Please specify other 'protected time' activities.

2022 Radiation Oncology Workforce Census

Trainee: Registrar duties and service: Training and mentoring

175. In my opinion, the most difficult aspects of Radiation Oncology are: (please select all that apply)

- [ ] Trying to understand different cancer treatments
- [ ] Radiation treatment planning
- [ ] Dealing with patients when treatments fail
- [ ] Palliative care
- [ ] Managing patients having concurrent chemotherapy
- [ ] Adjusting to ways of thinking and approaching treatment that are different from previous medical training
- [ ] Maintaining currency in developments in general medicine
- [ ] Lack of prior knowledge about specialty before starting in the training program
- [ ] Pathology, including molecular aspects
- [ ] Anatomy
- [ ] Cultural safety
- [ ] Other (please specify)

176. It would be useful to have a mentor in my department or network to help with research ideas, support projects, and for general assistance:

Strongly disagree	Disagree	Neutral or unsure	Agree	Strongly agree
○	○	○	○	○

Would you like to be a mentor for other trainees? Please answer yes or no.

2022 Radiation Oncology Workforce Census

Trainee: Registrar duties and service: Clinical duties

177. What clinical duties and responsibilities do you have? (please select all that apply)

- [ ] Attending local clinics
- [ ] Attending peripheral clinics
- [ ] Dictation of treatment summaries and letters
- [ ] Inpatient care
- [ ] Treatment simulation and planning
- [ ] Educational presentations
- [ ] Teaching of junior staff
- [ ] Teaching of allied staff
- [ ] Teaching of medical students
- [ ] Performing medical procedures
- [ ] Other (please specify)

____

The Royal Australian and New Zealand College of Radiologists®

The Faculty of Radiation Oncology

2022 Radiation Oncology Workforce Census

Trainee: Registrar duties and service: Part-time

178. During your training, have you been or will you be-working part-time for a period of 12 months or longer?

◯ No

◯ Yes (please note duration)

179. If given the option, would you like to do some of your training in a part-time capacity (i.e. 12-months or more)?

◯ Yes

◯ No

2022 Radiation Oncology Workforce Census

Trainee: Challenges associated with training

180. Please indicate whether you consider the following options to be a great source of stress for you:

	Strongly disagree	Disagree	Neutral or unsure	Agree	Strongly agree
Balancing work and family responsibilities	○	○	○	○	○
Job demands	○	○	○	○	○
Training demands	○	○	○	○	○
Spending time in remote centres	○	○	○	○	○
Job prospects	○	○	○	○	○
Other (please specify below)	○	○	○	○	○

Other (please specify)

181. How strongly do you consider leaving the specialty?

Not at all	Slightly	Neutral or unsure	Strongly	Very strongly
○	○	○	○	○

Please add any comments to qualify your answer

182. Over the past year, I have had enough time to pursue interests outside of work:

Strongly disagree	Disagree	Neutral or unsure	Agree	Strongly agree
○	○	○	○	○

183. How satisfied are you with your job?

- ◯ Very satisfied
- ◯ Satisfied
- ◯ Neither satisfied nor dissatisfied
- ◯ Dissatisfied
- ◯ Very dissatisfied

184. Please rate your level of stress on:

	Not stressed at all	A little stressed	Moderately stressed	Very stressed	Extremely stressed
An average work day	◯	◯	◯	◯	◯
A peak work day	◯	◯	◯	◯	◯

2022 Radiation Oncology Workforce Census

Trainee: Theranostics

185. Do you plan to sub-specialise in theranostics?

- ◯ Yes
- ◯ No

If no, would you like the option of sub-specialising?

186. If yes what level of further training do you think would be appropriate

- ◯ Mentoring from experienced radiation oncologist/ nuclear physician within the workplace
- ◯ A one-year post exam Fellowship in theranostics
- ◯ A combined radiation oncology/theranostics training pathway
- ◯ Other

Other (please specify)

2022 Radiation Oncology Workforce Census

Trainee: Future plans

187. Will you be continuing your career in Radiation Oncology?

- ◯ Yes Q188
- ◯ No Q204
- ◯ Undecided Q188

The Royal Australian and New Zealand College of Radiologists®
The Faculty of Radiation Oncology

2022 Radiation Oncology Workforce Census

Trainee: Future plans

188. Do you plan on undertaking a Fellowship year after training?

- ◯ Yes Q189
- ◯ No Q190
- ◯ Undecided Q190

2022 Radiation Oncology Workforce Census

Trainee: Future plans

189. For what reasons would you undertake a Fellowship? (please select all that apply)

- [ ] To be more competitive in the job market
- [ ] Gain specific skills and expertise
- [ ] Required by the centre I would like to work at
- [ ] Pursue research interests
- [ ] Ease the transition to Consultant position
- [ ] Other (please specify)

2022 Radiation Oncology Workforce Census

Trainee: Future plans

190. Do you plan to sub-specialise?

- ◯ Yes
- ◯ No
- ◯ If yes, please list your intended sub-specialty (up to 3).

191. My future practice includes:

	Yes	No
I wish to have completed a higher degree during my training	◯	◯
I am enrolled in a higher degree program	◯	◯
I am interested in teaching and would like to be a DoT and/or an examiner	◯	◯
I would like to participate in College committee work	◯	◯
I am interested in clinical trials and would like to be part of a trial or lead one	◯	◯

2022 Radiation Oncology Workforce Census

Trainee: Future plans

192. I am interested in an academic career because: (please select all that apply)

- [ ] I have an interest in teaching
- [ ] I enjoy the academic environment
- [ ] I wish to practice in a large teaching centre
- [ ] I am interested in clinical research
- [ ] I am interested in future leadership or administrative opportunities
- [ ] I am not interested in an academic career Q193
- [ ] Other (please specify)

____

2022 Radiation Oncology Workforce Census

Trainee: Future plans

193. I am not interested in an academic career because: (please select all that apply)

- [ ] My primary interest is patient care
- [ ] I want to practice in a smaller centre
- [ ] There is too much politics/bureaucracy involved
- [ ] Writing manuscripts is too time consuming
- [ ] Prefer not to do research
- [ ] Other (please specify)

2022 Radiation Oncology Workforce Census

Trainee: Future plans

194. Are you involved with the Medical Rural Bonded Scholarship Scheme, the Bonded Medical Places Scheme, or any other scholarship or return of service program that requires a period of post Fellowship practice in a rural area?

- ◯ Yes
- ◯ No

195. I would like to get a job where I can pursue my sub-specialty clinical interest:

Strongly disagree	Disagree	Neutral or unsure	Agree	Strongly agree
◯	◯	◯	◯	◯

196. For the first 10 years post-training, do you wish to work part-time for a period of 12 months or more?

- ◯ Full-time only Q199
- ◯ Part-time Q197

2022 Radiation Oncology Workforce Census

Trainee: Future plans

197. What FTE do you anticipate?

198. For what reasons did you provide the answer you gave to the previous question? (please select all that apply)

- ☐ Maternity/parental leave
- ☐ Family commitments
- ☐ Lifestyle
- ☐ Other (please specify)

2022 Radiation Oncology Workforce Census

Trainee: Future plans

199. I intend to practice:

- ◯ In the city/town where I did the most of my training
- ◯ Away from where I did the most of my training
- ◯ Undecided or not concerned

200. I intend to practice:

- ◯ In an urban department
- ◯ In a rural department
- ◯ Combination of urban and rural practice
- ◯ Undecided or not concerned

201. Do you think you would rather work in public or private practice?

- ◯ Public
- ◯ Private
- ◯ Both

202. For what reasons did you provide the answer you gave to the previous question? (please select all that apply)

- ☐ Lifestyle
- ☐ Family commitments (e.g. raising a family)
- ☐ Income
- ☐ Patient population
- ☐ Research opportunities
- ☐ Job availability
- ☐ Teaching opportunities
- ☐ Other (please specify)

203. For the future, my major concerns are: (please select all that apply)

- [ ] Fellowship opportunities
- [ ] Job availability
- [ ] Being potentially forced to work in a rural centre
- [ ] Declining government resources
- [ ] Other (please specify)

The Royal Australian and New Zealand College of Radiologists®
The Faculty of Radiation Oncology

2022 Radiation Oncology Workforce Census

Trainee: Choices

Q208

204. Overall, I am satisfied with my choice of training Network:

Strongly disagree	Disagree	Neutral or unsure	Agree	Strongly agree
○	○	○	○	○

205. Overall, I am satisfied with my career choice:

Strongly disagree	Disagree	Neutral or unsure	Agree	Strongly agree
○	○	○	○	○

206. Had I been aware of a perceived oversupply in the workforce I would have reconsidered joining the training program:

Strongly disagree	Disagree	Neutral or unsure	Agree	Strongly agree
○	○	○	○	○

Please add any comments you feel may be appropriate

2022 Radiation Oncology Workforce Census

Career progression

We are interested in the transition period between training and specialist practice. Please only answer this question if you have Fellowship of the RANZCR (FRANZCR).

207. What happened to you after election to Fellowship (FRANZCR)? (Please select all that apply)

- ☐ Locum
- ☐ Fellow or Advanced Trainee - Australia, New Zealand or Overseas
- ☐ Became a consultant
- ☐ Unemployed

Please explain your answer (e.g. what period were you in a locum position; where did you complete Fellowships/Advanced Training)

The Royal Australian and New Zealand College of Radiologists®
The Faculty of Radiation Oncology

2022 Radiation Oncology Workforce Census

Trainee: Impact of COVID-19

*** 208. COVID-19 had a negative impact on my training:**

- ◯ Yes
- ◯ No

*** 209. COVID-19 delayed the date that I took exams:**

- ◯ Yes
- ◯ No

If yes, please specify if the delay was in phase 1 or phase 2 exam and by how long.

[ ]

210. COVID-19 influenced my decision around Fellowship position

- ◯ Yes
- ◯ No
- ◯ None of the above

If yes, please explain:

[ ]

211. COVID-19 influenced my decision future career (eg subspecialty, work location, work life balance etc.)

- ◯ Yes
- ◯ No

If yes, please explain:

[ ]

212. COVID-19 meant that I was redeployed outside of radiation oncology

- ◯ Yes
- ◯ No
- ◯ If yes, please specify for how long.

[ ]

2022 Radiation Oncology Workforce Census

Trainee and Fellow: Impact of COVID -19

213. During the COVID-19 crisis, how has your workload changed?

-40% 50% 140%

214. Did your employer cut your working hours?

- Yes
- No

If yes, please specify by how much (in hours).

215. Did your employer vary your salary?

- Yes
- No

If yes, please specify the percentage change in salary.

216. Have your working hours and terms of employment returned to normal since the start of the COVID-19 situation?

- Yes
- No
- Not at all

2022 Radiation Oncology Workforce Census

Trainee and Fellow: Impact of COVID -19

217. With regard to my personal working patterns:

My work patterns were essentially the same	
I spent more time at home but managed the same workload	
I travelled less often to external clinics/treatment centres but continued to manage the same workload remotely	
I travelled less often to external clinics/treatment centres and passed work onto colleagues	
I had to absorb extra workload due to unavailability of colleagues	
Other	

218. During COVID-19, waiting times for treatment in my department

- Decreased
- Stayed the same
- Increased

219. As a result of COVID-19 my practice has

- Remained unchanged
- I am more likely to prescribe shorter courses (hypofractionated)
- I am less likely to offer palliative treatments
- Other (please specify)

220. Please list any other major changes in practice that occurred due to COVID-19

The Royal Australian and New Zealand College of Radiologists®
The Faculty of Radiation Oncology

2022 Radiation Oncology Workforce Census

Concluding comments

221. If you have any other comments or concerns related to the questions in this survey, please enter them below.

The Royal Australian and New Zealand College of Radiologists®
The Faculty of Radiation Oncology

2022 Radiation Oncology Workforce Census

End of survey

Thank you for taking the time to complete the 2022 Radiation Oncology Workforce Census.

If you have any questions regarding the census, please contact:

Nishant Gupta
Analyst - Economics and analytics
Nishant.Gupta@ranzcr.edu.au
+61 2 9268 9758
